# Abdominal internal oblique muscle hematoma of an obese middle‐aged man induced by cough

**DOI:** 10.1002/jgf2.639

**Published:** 2023-08-21

**Authors:** Daisuke Fujimori, So Sakamoto

**Affiliations:** ^1^ Department of Emergency Medicine Asahi General Hospital Chiba Japan

**Keywords:** emergency medicine, family medicine

## Abstract

An obese middle‐aged man presented for left abdominal pain. CT scan with contrast medium revealed hematoma in the left abdominal internal oblique muscle.

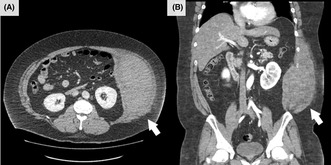

A 40‐year‐old man with a body mass index of 43 kg/m^2^ and three‐week history of productive cough presented to our hospital with left‐sided abdominal pain following a severe coughing episode. There was no history of trauma or respiratory disease. He was referred to our hospital due to persistent left‐sided abdominal pain.

Abdominal computed tomography (CT) with contrast medium revealed abdominal internal oblique muscle hematoma (160 × 160 × 80 mm in size) in the left side (Figure 1), and chest radiographs showed no abnormalities. There was no coagulopathy. After admission, the patient was conservatively managed using analgesics and abdominal compression bandages. Since the patient's vital signs remained stable, he was discharged the day after admission. The pain subsided gradually during the follow‐up sessions. A CT performed 10 days after pain onset confirmed reduction in the hematoma.

**FIGURE 1:**
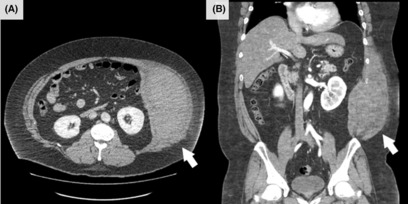
Contrast‐enhanced computed tomography of the abdomen showing the abdominal internal oblique muscle hematoma (arrow) (A: axial view, B: coronal view).

Cough is a reflex, which is vital for protecting the airway from secretions and foreign substances. However, strong cough can cause skeletal muscle complications.^1^ Abdominal wall hematomas can be caused by minor external forces, including cough. Moreover, most hematomas form within the rectus sheath. Internal oblique hematomas are very rare. Most abdominal wall hematomas occur after undergoing anticoagulation therapy.^2^ However, this patient was not administered anticoagulants. Weakness of the vessel wall due to obesity, advanced age, atherosclerosis, and pregnancy have been shown to be risk factors of abdominal hematoma.^3^, ^4^ There have been reports of internal oblique hematoma caused by cough as in this case,^4^ but none in a young man. Obese patients have increased number and size of blood vessels in the abdominal wall, and the reduced amount of fibrous tissue may have reduced the tamponade effect to stop bleeding.^5^ Paradoxically, the young man may have been affected by the greater muscular contraction of the abdominal wall. Symptoms of abdominal wall hematoma such as abdominal pain and mass and decreased hemoglobin levels are typical. However, these symptoms may be mistaken for tumors or inflammatory diseases of the abdomen.

Patients with abdominal wall hematomas are mostly treated conservatively with bed rest and analgesics. Invasive treatment is only indicated if hematoma enlarges or perforates into the abdominal cavity.

## CONFLICT OF INTEREST STATEMENT

None.

## INFORMED CONSENT

Written informed consent was obtained from the patient for publication of this clinical image.

## ETHICS APPROVAL STATEMENT

Ethical approval by the institutional review board of Asahi General Hospital, was not required at the authors' institution for this case report.

## References

[1] Irwin RS . Complications of cough: ACCP evidence‐based clinical practice guidelines. Chest. 2006 Jan;129(1 Suppl):54S–8S. 10.1378/chest.129.1_suppl.54S 16428692

[2] Cherry WB , Mueller PS . Rectus sheath hematoma: review of 126 cases at a single institution. Medicine (Baltimore). 2006 Mar;85(2):105–10. 10.1097/01.md.0000216818.13067.5a 16609349

[3] Hatjipetrou A , Anyfantakis D , Kastanakis M . Rectus sheath hematoma: a review of the literature. Int J Surg. 2015 Jan;13:267–71. 10.1016/j.ijsu.2014.12.015 25529279

[4] Kodama K , Takase Y , Yamamoto H , Noda T . Cough‐induced internal oblique hematoma. J Emerg Trauma Shock. 2013 Apr;6(2):132–4. 10.4103/0974-2700.110789 23723625 PMC3665063

[5] Goldstein JM , Sebire D . Abdominal wall haematoma in the obese: a dangerous phenomenon. J Surg Case Rep. 2013 Jul 23;2013(7):rjt060. 10.1093/jscr/rjt060 24964461 PMC3813510

